# A Statistical Framework to Interpret Individual Response to Intervention: Paving the Way for Personalized Nutrition and Exercise Prescription

**DOI:** 10.3389/fnut.2018.00041

**Published:** 2018-05-28

**Authors:** Paul A. Swinton, Ben Stephens Hemingway, Bryan Saunders, Bruno Gualano, Eimear Dolan

**Affiliations:** ^1^School of Health Sciences, Robert Gordon University, Aberdeen, United Kingdom; ^2^Applied Physiology & Nutrition Research Group, Rheumatology Division, Faculdade de Medicina, Universidade de São Paulo, São Paulo, Brazil; ^3^Institute of Orthopaedics and Traumotology, Faculty of Medicine, University of São Paulo, São Paulo, Brazil

**Keywords:** measurement error, biological variability, individual response, typical error, meaningful change, responders

## Abstract

The concept of personalized nutrition and exercise prescription represents a topical and exciting progression for the discipline given the large inter-individual variability that exists in response to virtually all performance and health related interventions. Appropriate interpretation of intervention-based data from an individual or group of individuals requires practitioners and researchers to consider a range of concepts including the confounding influence of measurement error and biological variability. In addition, the means to quantify likely statistical and practical improvements are facilitated by concepts such as confidence intervals (CIs) and smallest worthwhile change (SWC). The purpose of this review is to provide accessible and applicable recommendations for practitioners and researchers that interpret, and report personalized data. To achieve this, the review is structured in three sections that progressively develop a statistical framework. Section 1 explores fundamental concepts related to measurement error and describes how typical error and CIs can be used to express uncertainty in baseline measurements. Section 2 builds upon these concepts and demonstrates how CIs can be combined with the concept of SWC to assess whether meaningful improvements occur post-intervention. Finally, section 3 introduces the concept of biological variability and discusses the subsequent challenges in identifying individual response and non-response to an intervention. Worked numerical examples and interactive Supplementary Material are incorporated to solidify concepts and assist with implementation in practice.

## Introduction

It is widely recognized that traditional group intervention-based studies that focus on mean response are limited in the context of personalized sports nutrition, and that across populations, large inter-individual variability exists in response to health and performance related interventions. This variation occurs due to a myriad of factors, including an individual's genotype, phenotype, training status, and nutritional intake (1, 2). Accordingly, an increasing number of investigations are attempting to interpret individual data and classify participants as responders or non-responders to nutrition or exercise based interventions (3–11). In order to accurately interpret individual data collected from group-based interventions it is essential that researchers and practitioners consider a range of concepts including the confounding influence of *measurement error* and *biological variability*. In addition, the ability to interpret practical and statistical significance are enhanced by concepts such as *smallest worthwhile change* (SWC) and *confidence intervals* (CIs). The aim of this review is to describe a statistical framework that can be used by researchers and practitioners in the fields of applied sports nutrition and exercise physiology. The review is structured into three sections that build upon each other and develop into a coherent statistical framework. The initial section introduces concepts from classical test theory (12), namely measurement error, and describes how the calculation of typical error and the application of CIs can be used to express uncertainty in baseline values. Section two of the review builds upon the previous section and demonstrates how CIs can be combined with the concept of SWC to assess whether meaningful changes have occurred following an intervention. The final section of the review discusses the concepts of individual response and non-response and describes how the statistical framework developed can be used to estimate the proportion of response in a group-based intervention.

**Figure 1:**
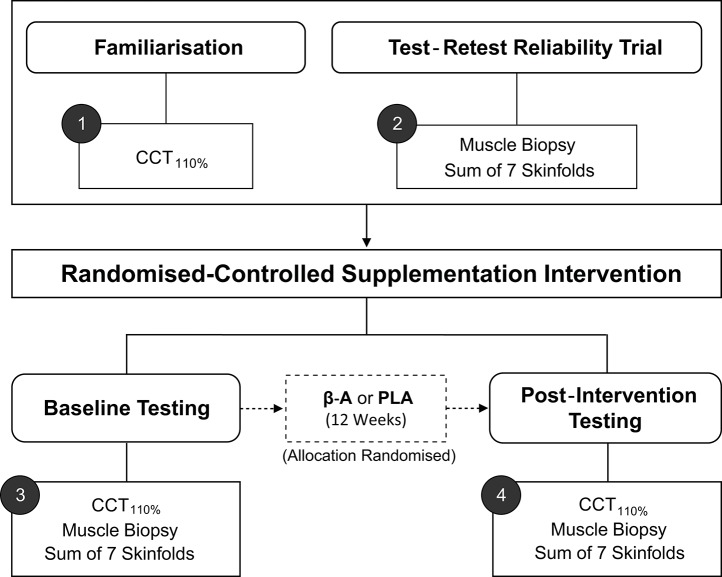
Schematic of hypothetical study design. CCT_110%_, High-intensity cycling capacity test; B-A, Beta-alanine supplementation; PLA, Placebo.

Key terms that will be used throughout the review have been defined in Table 1 and are italicized on first use. To facilitate understanding, worked examples are included throughout the review, from a hypothetical randomized controlled study (*n* = 20) investigating the influence of 12 weeks of beta-alanine supplementation on: (1) body composition [assessed by sum of 7 skinfolds]; (2) muscle carnosine content [assessed by high-performance liquid chromatography; HPLC], and (3) high-intensity cycling capacity [assessed by the CCT_110%_, a time-to-exhaustion test]. The study design is illustrated in Figure 1. Mock data from the study along with all worked examples are included in the accompanying supplementary digital file (SF). Automated spreadsheets are also included for readers to incorporate their own data sets and follow the procedures described within this review.

**Table 1:** Definitions of key terms.

**Term**	**Definition**
True score	A hypothetical value representing the score on a test that would be achieved if there were no measurement error.
Measurement error	Processes that causes an observed score on a test to be different from the true score. Measurement error comprises instrumentation and/or biological noise.
Observed score	The recorded value from a test, which comprises the true score, along with measurement error.
Instrumentation noise	Measurement error caused solely by the measurement apparatus, while true score remains unchanged.
Biological noise	Measurement error caused by biological processes (such as circadian rhythm, nutritional intake, sleep or motivation), while true score remains unchanged.
Typical error	The standard deviation of observed scores in repeated tests where true score remains unchanged.
Confidence interval	An interval that provides a range of plausible values for quantities that must be estimated (for example, true score) given the observed data.
Biological variability	Non-intervention related processes that cause true scores to change.
Smallest worthwhile change	A reference value selected by a practitioner or researcher to indicate a value beyond which a change in true score is likely to be meaningful in practice.
Response	Occurs when change in true score directly attributable to an intervention exceeds the smallest worthwhile change.

## 1. Establishing plausible baseline values (true score)

Practitioners and researchers routinely select and evaluate interventions depending on baseline information collected from an individual. Therefore, it is essential to consider the accuracy of baseline information and account for error in any decision-making process. An individual's *true score* can be viewed as their current stable level in the test of interest. In practice, we can never know an individual's true score as all measurement incorporates error and therefore, a single measurement from a test is simply referred to as an *observed score*. In classical test theory, it is assumed that if it were possible to conduct a large number of tests on the same individual then the values observed would follow a normal (Gaussian) distribution, with mean equal to the true score and standard deviation (σ) describing variability around this mean (12). In mathematical notation, we state that the observed score (*O*_*s*_) comprises a hypothetical true score (*T*_*s*_) and measurement error (ϵ), such that *O*_*s*_ = *T*_*s*_+ϵ (13). This perspective has clear implications when using baseline measurements to select interventions as an individual's true score always remains unknown. For tests that frequently produce large measurement errors, there is greater likelihood that observed scores will differ substantially from the true score, such that conclusions drawn, and interventions adopted may be unnecessary, ineffective, or indeed inappropriate.

Measurement error associated with any test comprises two primary sources, namely *instrumentation noise*, and *biological noise*. Here, we define instrumentation noise as error caused solely by the measurement apparatus. For example, offsets in calibration or variation in saddle position may cause observed performances in a cycling-based test, such as the CCT_110%_ to differ from the individuals true score (14). In contrast, we define biological noise as error in observed scores caused by biological processes, including, but not limited to, phenomena such as circadian rhythm, nutritional intake, sleep and motivation (1). When selecting and administering tests, every effort should be made to minimize the magnitude of measurement error. This can be achieved through adherence to standardized set-up, calibration and testing protocols, along with standardization of external factors likely to impact test scores through the introduction of additional biological noise (e.g., time of testing, nutritional intake and activity performed prior to testing). It is important to acknowledge, however, that these processes can only serve to reduce, but never to eradicate, measurement error.

**KEY POINTS**:

- *Due to the presence of measurement error, an individual's true score representing their current ability in a test always remains unknown and can only be estimated*.
- *Observed scores comprise the hypothetical true score and measurement error due to instrumentation and biological noise*.

### 1.1. Calculating the typical error of a test

**Figure 2:**
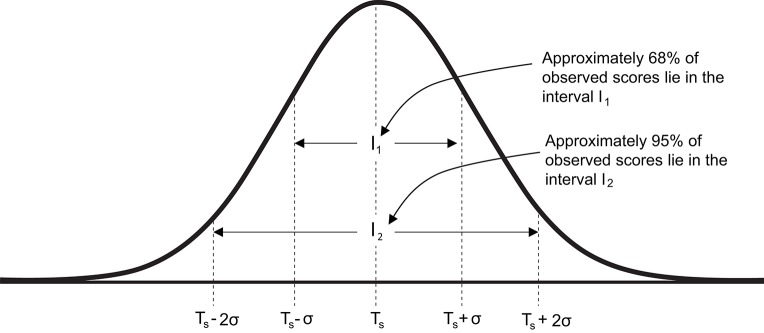
Graphical representation of the normal distribution of observed scores centered on true score. Ts, true score; σ standard deviation of repeated observed scores [also referred to as typical error (TE)].

As all observed measurements include error, it is important to estimate the potential magnitude of this error and thereby quantify uncertainty in any single measurement. Based on the assumption that observed scores follow a normal distribution centered on the true score, ~68% of observed scores lie in the interval *T*_*s*_ ± σ and ~95% of observed scores lie in the interval *T*_*s*_ ± 2σ (Figure 2). Therefore, the key to quantifying likely measurement error and ultimately providing ranges for true scores consistent with the data, requires estimation of the standard deviation (σ) for repeated tests. In applied physiology literature, this standard deviation is commonly referred to as the *typical error* (15) (TE), and from this point forward we will use the notation TE in all formulae instead of σ.

Two primary methods are available to estimate the TE of a test, including: (1) multiple repeated tests performed by a single individual; or (2) a single test-retest performed by a group of individuals. Using the first approach, the TE is estimated by calculating the standard deviation of observed scores obtained from a single individual performing multiple tests within a time-frame whereby the true score remains theoretically stable. Suitable time-frames will depend on the specific characteristics of a given test. For example, true score in the CCT_110%_ is largely dependent on the capacity of the cardiovascular and muscular systems, neither of which are likely to undergo substantial physiological changes in the absence of intervention within short time-frames. The true score for CCT_110%_ performance should therefore remain stable across days or even weeks, although biological noise in particular (e.g., motivational factors), may cause observed scores to fluctuate within this time-frame (16). The accuracy of the TE estimate based on repeated tests conducted with an individual will generally increase with the number of repeated tests but may require more than 10–20 tests to obtain suitable accuracy. This requirement presents logistical difficulties in relation to resources required to repeatedly administer many tests with any one individual. In addition, even if these logistical difficulties could be overcome, the testing process itself may lead to a change in the individual's true score, and as a result, the estimate of the TE will become inflated (15). Continuing the CCT_110%_ example, repeated performance of a high-intensity activity to exhaustion is likely to create a strong stimulus for adaptation (17), thereby causing true score to change and estimates of variation to subsequently increase. For these reasons, single individual approaches to estimate TE are rarely used in the exercise sciences.

Based upon the aforementioned limitations, the most popular method to estimate the TE of a test relies on multiple individuals each performing a single test-retest assessment (15). This approach is well suited to those working with sport teams or groups taken from a relatively homogenous population. Group based calculations for estimating TE generally rely on the assumption that, whilst true scores may vary between individuals, TE is consistent across the population being assessed. Based on this assumption, if test-retest values are obtained from a group of individuals over a period where true scores are expected to remain constant, TE can be estimated from the difference scores obtained. Difference scores are calculated for each individual in the group test-retest by simply subtracting the observed scores in test 1 from the observed scores in test 2 (i.e., observed[test2]—observed[test1]). These difference scores are described by a normal distribution with mean 0 and standard deviation equal to $\sqrt{2}$TE. The standard deviation is equal to $\sqrt{2}$TE as the variance (which is equal to the square of the standard deviation) of the difference scores is equal to the variation in test 1 plus the variation in test 2 (i.e., TE^2^ + TE^2^ = 2TE^2^). Therefore, to obtain the TE estimate with a group test-retest design, we first calculate the difference score for each individual, calculate the standard deviation of the differences scores, then divide this value by $\sqrt{2}$. Formulae and worked examples of this TE calculation are included in the Supplementary File (SF-S2) using test-retest data from sum of skinfolds and muscle carnosine biopsies conducted 48–72 h apart (i.e., a time frame where true score is unlikely to change). For the muscle carnosine data, the standard deviation of the difference scores from the repeated tests was calculated to be 0.74 mmol·kg^−1^dm; hence the estimate of TE is $0.74/\sqrt{2}$ = 0.52 mmol·kg^−1^dm. It is important to note that this calculation represents an estimate of TE and is unlikely to exactly match the real value. Therefore, we use the notation $\hat{\text{TE}}$ from this point forward in calculations where we refer to an estimate of TE.

**KEY POINTS**:

- *Typical error represents the variation in observed scores caused by measurement error when an individual performs repeated tests*.
- *An* ***estimate*** *of typical error can be obtained by calculating the standard deviation of repeated tests performed by a single individual, or more commonly using test-retest data from a group over time periods where true scores are not expected to change*.

#### 1.1.1. True score confidence intervals

Once an observed score and TE estimate have been obtained, a *confidence interval* (CI) for the true score can be created. CIs are used to quantify uncertainty in estimates that cannot be directly measured (18). Therefore, calculation of a CI for a true score provides a range of plausible values given the observed data. We have highlighted that conceptually, true score is equal to the mean of a large number of non-interacting repeated tests, and that observed scores follow a normal distribution around this mean. Therefore, measurement error in a single observed score is just as likely to be positive as it is negative. Therefore, true score CIs are created by adding and subtracting a multiple of the estimated TE to the observed score, with larger multiples creating wider intervals (Figure 2). CIs are interpreted as a property of a procedure and when used repeatedly, the percentage of intervals calculated that include the true value being estimated will match the % CI used (19). In other words, if a practitioner routinely follows the procedure of estimating TE and calculating, say, 95% CIs for true scores, then through maintaining a compilation of these values over their career the percentage of intervals that contain the true score will be ~95%. A key point here is that a CI based on a single dataset should not be interpreted probabilistically (19), as it is possible to obtain a very high, or very low estimate of TE by chance, such that true score CIs calculated will be inappropriate.

#### 1.1.2. Calculating true score confidence intervals of different widths

The measurement assumptions outlined in the previous section enable practitioners to calculate various CI widths by multiplying their TE estimate by values that are based on the normal distribution. In the first row of Table 2, we provide the values required to obtain a range of standard CI widths. Returning to our muscle carnosine example where we obtained a TE estimate of 0.52 mmol·kg^−1^dm, an approximate 95% true score CI for an individual with an observed score of 11.3 would equal 11.3 ± (1.96 × 0.52) = (10.3−12.3) mmol·kg^−1^dm. It is important to note that the values provided in the first row of Table 2 provide only approximate CIs as the TE value describing variation of repeated performances is unknown and only the estimate $\hat{\text{TE}}$ is available. The accuracy of this estimate depends primarily on the number of individuals (or number of repeated trials) used in the test-retest calculation. CIs based on a TE estimate from smaller samples sizes (e.g., 6–12 participants) will in general, be less accurate than those based on larger sample sizes (e.g., >30). To account for this additional uncertainty, the value used to multiply $\hat{\text{TE}}\;$and obtain a given CI should be adjusted and a larger multiple used. In the subsequent rows of Table 2, we present adjusted values that provide more accurate CIs over a range of test-retest sample sizes. Values presented in Table 2 clearly illustrate that for CI widths close to 50%, the sample size used to estimate TE has minimal effect on the multiple required. However, for wider CIs such as a 95% CI, adjustment for smaller test-retest sample sizes can result in more notable differences. In our previous example (*n* = 20), the individuals 95% true score CI for muscle carnosine was 10.3−12.3 mmol·kg^−1^DM. In contrast, if $\hat{\text{TE}}\;$was obtained from test-retest with only 5 individuals, the adjusted 95% CI would equal 11.3 ± (2.78 × 0.52) = 9.9−12.7 mmol·kg^−1^dm. To identify the number of individuals required for a test-retest, the values presented in Table 2 can provide insight. Practitioners can make an initial estimate of TE and create for example, 95% true score CIs adjusting for *n* = 5, 10, 20, 30, and 50. Interpreting the practical relevance of the different CI widths generated can then be used to inform sample size used. For readers that require more detail on the adjustment approach a full explanation of how to obtain the values for any sample size and CI width is presented in Appendix 1. We also present in the Supplementary File an interactive calculator to calculate unadjusted and adjusted CIs of different widths, that can be combined with the mock data set (SF-S4) or a reader's own data set (SF-S5).

**KEY POINTS**:

- *Confidence intervals can be used to present plausible values of an estimate given the observed data. Repeated application of estimating typical error and associated confidence intervals will result in a match between the percentage of intervals containing the true value and the percentage interval adopted*.
- *True score confidence intervals can be calculated using the observed score and a multiple of the estimated typical error. The multiple selected depends on the desired width of the confidence interval and the number of individuals (or number of repeated tests) used to estimate the typical error*.

**Table 2:** Typical error multiples required to calculate confidence intervals of different widths (non-adjusted and adjusted for sample size).

**Confidence interval width**	**50%**	**60%**	**70%**	**75%**	**80%**	**85%**	**90%**	**95%**	**99%**
TE multiple non-adjusted	0.67	0.84	1.04	1.15	1.28	1.44	1.64	1.96	2.58
TE multiple adjusted (*n* = 50)	0.68	0.85	1.05	1.16	1.30	1.46	1.68	2.01	2.68
TE multiple adjusted (*n* = 30)	0.68	0.85	1.06	1.17	1.31	1.48	1.70	2.05	2.76
TE multiple adjusted (*n* = 20)	0.69	0.86	1.07	1.19	1.33	1.50	1.73	2.10	2.86
TE multiple adjusted (*n* = 10)	0.70	0.88	1.10	1.23	1.38	1.57	1.83	2.26	3.25
TE multiple adjusted (*n* = 5)	0.74	0.94	1.19	1.34	1.53	1.78	2.13	2.78	4.60

### 1.2. Literature based confidence intervals

**Figure 3:**
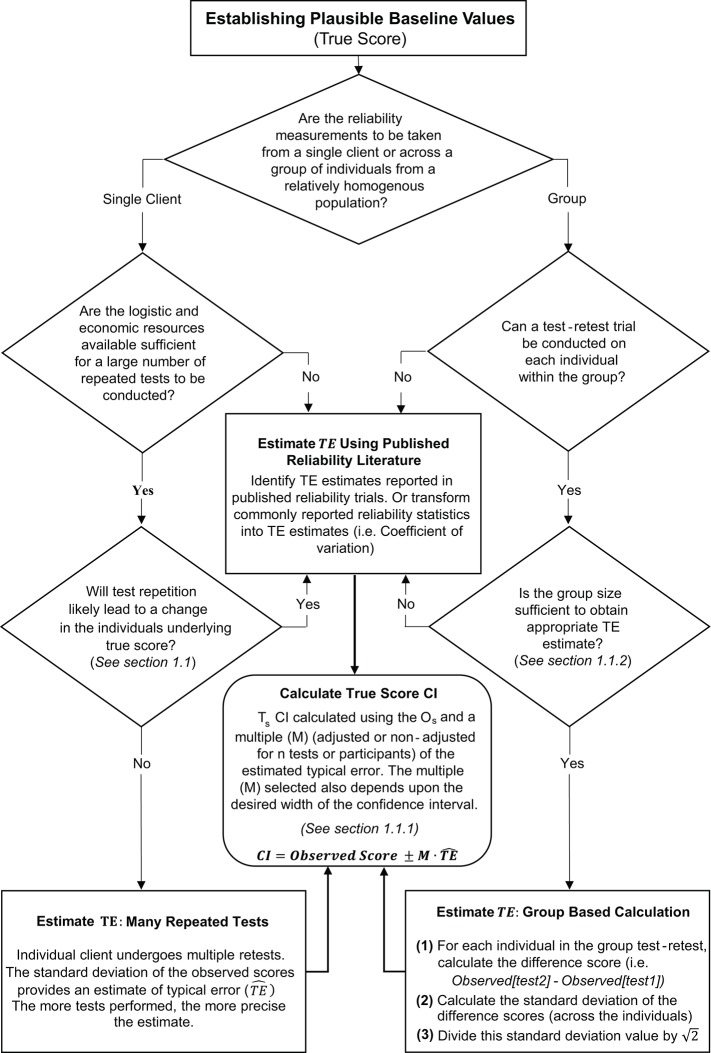
Schematic overview of procedures to estimate typical error and calculate true score confidence intervals.

In circumstances where it is not feasible to perform repeated measurements on a single individual or group, practitioners can create CIs for true scores using reliability data published in the literature. To obtain accurate CIs it is recommended that practitioners source reliability data collected using the same test protocols employed with their own clients, and that the populations match as close as possible. TE estimates are commonly reported in reliability studies and practitioners can directly use these published values to calculate CIs using the methods described in Section 1.1.1. It is also common for researchers to report other reliability statistics that can be transformed into a TE estimate. One commonly reported reliability statistic that can easily be transformed is the coefficient of variation (CV). The coefficient of variation is a percentage that expresses the size of the TE relative to the mean [*CV%* = (*TE*/*mean*) × 100; (20)]. Therefore, a true score CI can be obtained using published CV values by first identifying the TE estimate from $\hat{\text{TE}}=\left(CV\times O_{s}\right)/100$, then applying the procedures outlined in Section 1.1.1. In our hypothetical study, duplicate measurements were not available for the CCT_110%_, and therefore we describe here (and in Supplementary File: SF-S6) how to estimate a TE and true score CI based on previously published data. Saunders et al. (21) reported that the CV for total work done in the CCT_110%_ was 4.94% (21). Therefore, for an individual with an observed score of 43.0 kJ, we calculate an estimated TE of (4.94 × 43.0)/100 = 2.1 kJ. Using the values in Table 2, we can calculate a range of true score CIs. If we select, say, an unadjusted 75% CI then we would obtain 43.0±(1.15 × 2.1) = 40.6−45.4 kJ. An overall summary of the process for estimating TE and calculating true score CIs is presented in Figure 3.

**SUMMARY OF KEY POINTS FROM SECTION 1.2**:

- *Confidence Intervals can be calculated from literature using published TE values or other reliability statistics (e.g., coefficient of variation (CV))*.

## 2. Assessing whether meaningful changes have occurred post-intervention

As described in the previous section, an individual's true score cannot be known due to the existence of measurement error and this uncertainty must be accounted for when interpreting pre- to post-intervention change. This requirement is particularly relevant in sports nutrition based interventions where improvements are often small in magnitude whilst many performance based outcome measures may be prone to relatively large measurement errors. For example, Jeukendrup et al. (22) showed that a time-to-exhaustion test at 75% of previously determined maximal power output had a CV of 26.6%, which is far in excess of the 5-15% changes in exercise capacity shown with beta-alanine supplementation (23–26). In Section 1.1.2, we used CIs to express our level of uncertainty in baseline test scores. Similarly, CIs can be used to express the level of uncertainty in the change in test scores due to an intervention (*true score change*). Many of the tools and calculations that were introduced in the previous section on baseline scores are also relevant when considering appropriate methods to quantify and interpret change across an intervention. In the following sections we describe minor alterations required to calculate CIs for true score change in comparison to baseline true score. We also introduce the concept of SWC to better assess the effectiveness of an intervention.

### 2.1. Confidence intervals for true score change

If we assume that measurement error of a test is not only consistent across individuals in a group, but also consistent for individuals across an intervention, then observed scores will display the same variation around the true pre-intervention score and the true post-intervention score. It follows that observed change scores (*OS*_*post*_ − *OS*_*pre*_) are described by a normal distribution with mean equal to the true score change and standard deviation (i.e., standard deviation of the change scores) equal to $\sqrt{2}$TE. Note, this is the same result discussed in section 1 for the test-retest situation, except here we expect true scores to change due to the intervention. Therefore, to estimate this standard deviation we simply take our previous TE estimate (obtained from repeated tests on a single individual, test-retest for a group, or from published literature) and multiply by $\sqrt{2}.$ CIs are then obtained using the procedures outlined in section 1.1.2, except here we apply our estimate around the observed difference across the intervention. For example, in our hypothetical data set, the TE for the muscle carnosine content analysis was 0.52 mmol^.^kg^−1^DM, with participant 8 (from the beta-alanine group) displaying an observed change score (difference pre-post) of 4.37 mmol·kg^−1^DM. For this example, we will calculate an unadjusted 50% true score change CI using the appropriate multiplier presented in Table 2. The required calculation is therefore $(OS_{post}-OS_{pre})\pm \left(0.67\times \sqrt{2}\hat{\text{TE}}\right)=4.37\pm \left(0.67\times \sqrt{2}\times 0.52\right)=3.9-4.9$ mmol^.^kg^−1^DM. Interactive true score change CI calculators are provided in the Supplementary File for the study mock data (SF-S7) and the readers own data (SF-S8).

**SUMMARY OF KEY POINTS FROM SECTION 2**:

- *True score change occurs whenever the underlying stable characteristic measured by a test changes*.
- *The estimated typical error can be used to create confidence intervals for true score change pre- to post-intervention*.

### 2.2. Criteria for assessing meaningful changes and the smallest worthwhile change

**Figure 4:**
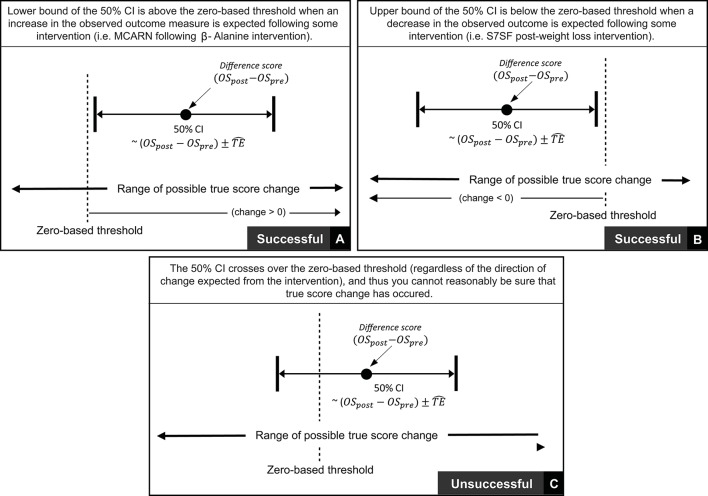
Interpretation of true score change confidence intervals using zero-based thresholds.

In the previous section we described procedures to calculate true score change CIs that provide a range of plausible values given the data observed. In practice, it is recommended that interventions are classified as successful or not for each individual based on whether CIs for true score change lie within a pre-defined region (27). If for example, a practitioner deems that *any* true score change in the desired direction is to be regarded as meaningful, then an intervention for an individual would be classified as successful if both ends of the true score change CI lie to the desired side of the zero line (see Figures 4A,B). It has also been suggested that CIs for true score change be calculated based on the observed change plus/minus the estimated TE ($OS_{post}-OS_{pre}\pm \hat{\text{TE}}$; (27)). This simple calculation provides a close approximation for a 50% true score change CI ^*^^1^. Additionally, with the assumption that observed score change provides a non-biased estimate, we should expect on 50% of occasions for the true score change to lie within the calculated interval, on 25% occasions the true score change to lie below the interval, and on 25% of occasions the true score change to lie above the interval. As a result, if interventions are deemed a success only if observed score change $\pm \;\hat{\text{TE}}$ lie to the desired side of the 0 line, then the proportion of times an intervention will correctly be identified as a success will be greater than 75% in the long-run. The accuracy of this statement is illustrated in Figure 4 using the most conservative successful case, where the approximate 50% CI bound lies on the zero line. Detailed calculations of this process are presented in the Supplementary File (SF-S8), however, we briefly provide an example here. In our hypothetical data set, participant 4 recorded an observed decrease of 7.1 mm in the sum of 7 skinfolds pre- to post-intervention. Additionally, the TE estimate obtained for sum of seven skinfolds from the hypothetical data was 1.35 mm. Therefore, we calculate the approximate 50% true score change CI with −7.1 ± 1.35 = (−8.45 to −5.75) mm. As both the upper and lower bound of the interval are negative (which is the desired direction indicating a reduction in body fat), we conclude that the intervention was successful for this participant.

**Figure 5:**
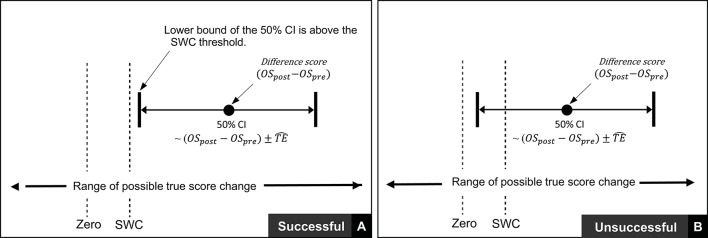
Interpretation of true score change confidence intervals using smallest worthwhile change.

Thus far in this section, we have focused on scenarios where any true score change greater than 0 in the desired direction is considered meaningful. In many research settings, this approach will be appropriate, given that researchers are likely to deal with experimental scenarios and unknown outcomes. In contrast, in other situations, researchers and practitioners may implement interventions whereby relatively large improvements are expected, such that more substantive changes are required in order to classify an intervention as a success. Take for example our hypothetical intervention, which aims to increase muscle carnosine content through beta-alanine supplementation. Previous investigations indicate that 4 weeks of supplementation can increase muscle carnosine content by 40–60% (28) and more recently, maximal increases ranging from 60 to 200% have been reported for participants supplementing for 24 weeks (26). Increased intramuscular carnosine content causes a subsequent increase in intramuscular buffering capacity, which may counteract high-intensity induced acidosis and thus fatigue (29). Given this context, establishing that a participant experienced a true score increase in intramuscular carnosine just beyond zero would be considered practically meaningless, given the negligible influence on buffering capacity and subsequent high-intensity exercise performance. Instead, researchers and practitioners may choose to identify threshold values beyond zero that represent the smallest change required to be practically relevant. This threshold value is generally referred to as the SWC and is often selected subjectively by practitioners based on what they believe is practically relevant or from their experience working with a particular client base using similar interventions. Alternatively, calculations of effect size (e.g., Cohen's D) can be used to objectively determine the SWC, with a value of 0.2 times the baseline between-individual standard deviation often considered to be appropriate (27). Approaches to calculate SWC are discussed in detail elsewhere (30–32). Once a SWC value has been selected, the general approach to determining meaningful change post intervention remains the same and for each participant an intervention is classified as a success if the true score change CI lies beyond the selected SWC (Figure 5A, with non-success illustrated Figure 5B).

To effectively implement these procedures, tests that comprise appropriate measurement error relative to the SWC are required. It is recommended when implementing this approach that an *a priori* determination of the SWC deemed practically relevant is made. In most cases, it would be expected that the majority of individuals that engage in an intervention should exceed the SWC, therefore, the threshold set should be below the likely projected change for most individuals. An appropriate test with regards to measurement error would then be one where the TE is no larger than the gap between these two values (namely expected change and SWC). As an example from the hypothetical study considered here, a practitioner may decide to set a SWC threshold for the CCT_110%_ by multiplying the baseline between-participant standard deviation by 0.2 (i.e., a ‘small’ effect), providing a value of 1.6 KJ. From previous research, 4 weeks of beta-alanine supplementation has been shown to improve CCT_110%_ performance by ~10–15% (23–25). If we consider participant 1 from the supplementation group with a baseline value of 42.8 kJ and expect a realistic 10% improvement, then we project a value of 47.1 kJ by the end of the intervention equalling an improvement of ~4.3 kJ. Given a TE value of 2.2 kJ and implementing the recommended process, in order to judge the intervention successful, participant 1 would require an observed score improvement of at least 1.6 + 2.2 = 3.8 kJ (which exceeds the *a priori* set SWC but is also less than what we expect in general). In contrast, if the estimated TE was considerably higher (e.g., due to lack of control for instrumentation or biological noise), say 5 kJ, then the same participant would require an observed score improvement of at least 1.6 + 5 = 6.8 kJ, which is a larger change than the literature indicates would typically be expected. This approach may therefore frequently lead to interventions being deemed not-successful when in fact the large TE may have masked any detectable improvement. In situations such as this, consideration of factors which may reduce the TE of the test are advised (e.g., enhanced standardization of procedures, repeat familiarisations), however if this is not possible, then this particular test may lack the sensitivity required to detect meaningful changes, and an alternative one may be required. Worked examples of this entire process with mock data are presented in the Supplementary File (SF-S9) along with an interactive calculator that can be used to identify successful interventions with readers own data (SF-S10).

**KEY POINTS**:

- *When assessing the effectiveness of an intervention, it is recommended that practitioners identify a smallest worthwhile change (which in some cases may be zero)*.
- *Practitioners may choose to judge an intervention successful for an individual if the observed score change (post-pre)* ± *typical error lie beyond the smallest worthwhile change*.
- *Practitioners must ensure that tests include typical error values that are not too large such that individuals require unrealistic improvements for confidence interval bounds to lie beyond smallest worthwhile change values*.

## 3. Response to intervention and the role of biological variability

Throughout sections 1 and 2 we described procedures to quantify the level of uncertainty in baseline values, quantify the level of uncertainty in change across an intervention, and to identify if observed changes represent meaningful improvements. These procedures outlined do not, however, identify whether underlying changes occurred as a direct result of the intervention or as a result of unrelated confounding factors. Across time periods reflecting those typically used for chronic supplementation or training interventions, it is possible that an individual's true score may change due to factors external to the intervention. Take for example our 12 week hypothetical study, where CCT_110%_ was used to assess cycling capacity. High-intensity exercise performance is influenced by a wide range of factors, including nutritional intake, chronic sleep patterns and physical activity levels, with 12 weeks providing sufficient time for true scores to change in response to alterations in any of these factors. We refer to these intervention independent causes of change as *biological variability*. When combining this concept with measurement error, the potential challenges in identifying if a single individual has accrued meaningful improvements as a direct result of an intervention become clear. In section 2 of this review we outlined procedures that can be used to judge whether meaningful changes were likely to have occurred. However, these procedures do not determine the extent to which changes were the direct result of the intervention or effectively “random” external causes. For this reason, we recommend that individual responders (those that experience meaningful changes due to the direct effects of an intervention) and non-responders (those that do not experience meaningful changes due to the direct effects of an intervention) be considered as theoretical constructs that can never truly be known. Additionally, intra-individual variation in response to an intervention is rarely considered. For example, even if it were possible to establish that an individual's true score had not changed due to direct effects of an intervention (to be accurately labeled as a non-responder), it does not hold that the same result will occur if the intervention is repeated at a later time, or more conceptually, that this would be the case in each instance were it possible for the individual to complete the intervention on many occasions simultaneously. Indeed, inconsistent intra-individual changes to the same sodium bicarbonate based intervention have previously been reported, with individual analysis showing only 1 out of 15 participants improved on all four occasions above the normal variation of the test, whereas 9 out of 15 improved on at least one occasion (10). Consequently, the term *response* (and *non-response*) is preferred to indicate that in a single instance of a particular intervention, an individual has experienced (or not) a true score change caused directly by the intervention that exceeds the SWC. Given all the challenges that exist in identifying if an individual has responded to an intervention with only a small number of data points, we concur with recent recommendations (33, 34) that researchers focus on identifying the proportion of response in group-based interventions (discussed in the following sections) or attempt to identify factors associated with response/non-response (which is considered beyond the scope of this particular review, with readers referred to Hopkins (34) for further discussion).

In the remaining sections of this review we describe procedures in group-based interventions to estimate variability in true score change directly attributable to the intervention, and, subsequently, to estimate proportion of response in a group. The procedures outlined are required during interventions with periods long enough for true score change to occur as a result of biological variability. In contrast, many nutritional supplements (e.g., caffeine or sodium bicarbonate acutely function after a single dose (35, 36) and provided the repeated tests take place within a sufficiently short time-period, then consideration of measurement error alone may be sufficient to identify proportion of response and non-response.

**KEY POINTS**:

- *Non-intervention related factors can often cause true scores to change. Collectively, these factors are referred to as biological variation*.
- *The terms response and non-response are used to indicate whether an individual's true score change caused by the intervention alone exceeds the smallest worthwhile change, or not, respectively*.

### 3.1. Estimating variability caused by intervention

It is widely recognized that the most logical means of quantifying variability caused by an intervention is to include a control group or to use data from similar controls published in literature (2, 33, 34). Quantifying variation in change across a control group provides an assessment of both measurement error and biological variation. In contrast, variation in change experienced in an intervention group also accounts for the differential effects caused by the intervention. As a result, true score variation due to an intervention is equal to the change score variance of the intervention group minus change score variance of the control group. As with all concepts described in this review, variation is most useful when expressed as a standard deviation, and here we define the quantity of interest as the intervention response standard deviation (σ_*IR*_). In practice, this standard deviation is estimated with the following formula ${\hat{\sigma }}_{IR}=\sqrt{SD_{Int}^{2}-SD_{Con}^{2}}$, where $SD_{Int}^{2}$ is the square of the calculated standard deviation of the observed score change from the intervention group, and $SD_{Con}^{2}$ is the square of the calculated standard deviation of the observed change scores from the control group (34). Using data from our hypothetical study as an example, MCARN standard deviations of observed score change from the control and intervention group were found to be 1.24 and 5.22 mmol^.^kg^−1^DM. Note, this large difference in standard deviations measured between groups provides evidence that true change directly attributable to the intervention was highly variable across participants (33). We find that ${\hat{\sigma }}_{IR}=\sqrt{SD_{Int}^{2}-SD_{Con}^{2}}=\sqrt{5.22^{2}-1.24^{2}}=5.07$ mmol^.^kg^−1^DM, and as explained in the following section, this value can then be used to estimate the proportion of response.

**KEY POINTS**:

- *Variation in the effect of an intervention across individuals can be estimated by comparing the standard deviation of observed score change in an intervention and control group*.
- *The statistic that reflects variation in intervention effect across individuals is referred to as the intervention response standard deviation (*σ_*IR*_*)*.

### 3.2. Estimating proportion of response

**Figure 6:**
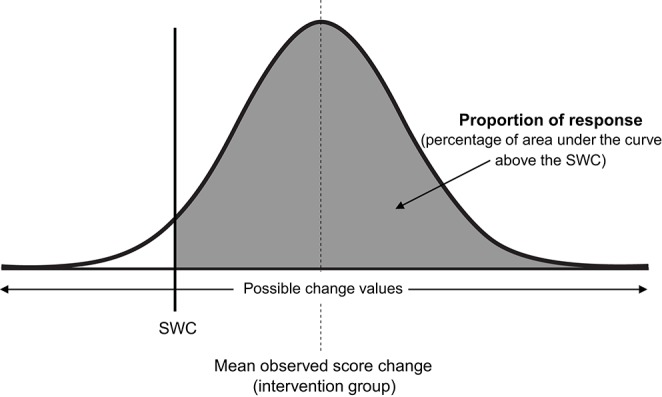
Proportion of intervention group response. SWC, smallest worthwhile change; σ_*IR*_, Intervention response standard deviation.

Consistent with all approaches used previously in this review to estimate quantities of interest (e.g., baseline true score, true score change, or here, proportion of response), we assume a normal distribution, such that true score change directly attributable to the intervention follows a normal distribution centered on the mean observed score change, with standard deviation equal to σ_*IR*_ (see Figure 6). As the total area of any probability distribution is equal to one, the estimate of the proportion of response is obtained by calculating the area of the derived normal distribution that lies beyond the SWC (Figure 6). A full example calculation covering this process is included in Supplementary File (SF-S11), along with automated spreadsheet where readers can estimate proportion of response for their own datasets (SF-S12). Here we continue our example using the muscle carnosine data from our hypothetical study to provide greater clarity. The mean observed score change from the intervention group across the twelve-week period was 10.20, and as calculated in the previous paragraph, ${\hat{\sigma }}_{IR}=5.07.$ Therefore, the true score change attributable to the intervention is modeled as a normal distribution with mean 10.20 mmol^.^kg^−1^DM and standard deviation 5.07. If we select a SWC from standard procedures by calculating 0.2 times the baseline standard deviation, we obtain a threshold value of 2.0 mmol^.^kg^−1^DM. Using the interactive calculator in the Supplementary File (SF-S11), we find that 0.947 of the area of the normal distribution described lies beyond the SWC and so we estimate that the proportion of individuals that responded to the intervention with regards to muscle carnosine content was 0.947 (i.e., we estimate that ~95% of the supplementation group responded). This value is only an estimate, that we expect will become more accurate with greater numbers in both the intervention and control groups due to better precision in estimating ${\hat{\sigma }}_{IR}.\;$Proportion of response is a more complex estimator than those encountered previously in the review, and confidence intervals are best derived through a resampling process such as bootstrapping. Briefly, in bootstrapping we treat our sample data as the population and repeatedly draw random samples (from the original data) each time carrying out the same calculation (e.g., proportion of response). Uncertainty in the estimate is then expressed by examining variation in the results obtained. In the Supplementary File we have included an automated spreadsheet to estimate proportion of response with the readers own data and to calculate selected confidence interval widths through bootstrapping (SF-S12). For the muscle carnosine data, the 95% CI for the proportion of response estimate was found to be 88.2–100%.

**KEY POINTS**:

- *The different effects of an intervention across individuals can be modeled as a normal distribution centered on the mean observed score change with standard deviation* σ_*IR*_.
- *Proportion of response is estimated by calculating the area of the normal distribution that lies beyond the smallest worthwhile change*.
- *Confidence intervals for the proportion of response estimate can be obtained through bootstrapping*.

### Summary and practical recommendations

Throughout this review, we have described procedures required to interpret data collected from individuals both pre- and post-intervention. Careful and deliberate procedures are required to interpret the data appropriately, due to the fact that all measurement incorporates some degree of error (measurement error = instrumentation noise + biological noise), and changes can often occur due to factors independent of the intervention (biological variability). The procedures we have outlined enable practitioners and researchers in the area of sports nutrition to (1) establish plausible baseline values; (2) assess whether meaningful changes have occurred after an intervention; and (3) estimate the proportion of individuals in a group-based intervention that responded/did not respond to the intervention. We conclude this review with a brief summary including practical recommendations.

Prior to conducting any intervention, practitioners and researchers require baseline data to direct their choice of intervention and provide initial values to monitor and assess an intervention's progress and effectiveness. Tests and measurement procedures adopted should seek to minimize measurement error, which includes both instrumentation and biological noise. It must be recognized, however, that even when the testing environment is controlled as much as possible, some degree of measurement error will always exist. Therefore, typical error should be calculated and CIs applied to baseline measurements to provide a range of plausible true scores given the data observed. Ideally, CIs should be calculated with reliability data obtained by the practitioner using the actual equipment and procedures implemented with their clients. However, where this is not feasible, it is recommended that practitioners obtain data from published reliability studies that match their own procedures as closely as possible with regards to testing protocols and participants.

In situations where CI widths are so wide as to provide no actionable baseline information, practitioners should re-consider the specific %CI used and consider whether this can be reduced given the context of the measurement. For example, 95% CIs frequently produce large ranges for true scores and practitioners have to consider whether they require the actual true score to reside within intervals calculated in 95% of occasions. Where the safety of a client is not influenced by the intervention, narrower %CIs can be justified. For example, practitioners may choose instead to construct CIs with the observed score plus/minus the estimated TE. This calculation is simple to create and maintain across spreadsheets that practitioners may create and for baseline scores provides approximate 70% true score CIs. However, if true score intervals calculated with similar %CIs still provide limited actionable information, this suggests that the test and/or measurement processes adopted create measurement errors too large to be of practical use, and therefore an alternative and more reliable test should be considered.

Once an intervention has been completed, it is good practice to estimate true score change and provide a CI to identify a range of plausible values given the observed data. Such CIs represent the all cause change across the intervention and do not distinguish between change caused by the intervention and external factors. Where appropriate, practitioners can identify the SWC deemed to be of practical relevance for the individual, with success judged to occur when the observed score change plus/minus the estimated TE lie beyond the threshold set. In research settings, the threshold value may be set at 0, however, practitioners should select this value *a priori*. Practitioners should ensure that the estimated TE is not so large that successful interventions will frequently be deemed not-successful. To ensure this is not the case, it is recommended that practitioners identify, for example, average observed changes for specific groups of clients (which should be larger than the SWC) and make sure that TE is smaller than the difference between the average change and SWC. Where this cannot be achieved, participants will in general, be required to obtain true score changes greater than average in order for interventions to be deemed successful.

The existence of biological variability renders it challenging to isolate true score change directly caused by the intervention. For this reason, we recommend that researchers interested in this area and limited to designs with infrequent data collection (e.g., pre-intervention and post-intervention), focus at the group level and estimate proportion of response rather than attempt to identify any one individual as a responder or non-responder, and where appropriate, attempt to identify factors associated with response/non-response [see Hopkins (34) for further discussion]. To estimate the proportion of response, a control group is required, with variation between control and intervention groups compared to quantify variation in true score change directly attributable to the intervention. An estimate of the proportion that responded can then be calculated by using the observed difference scores, standard deviations calculated from intervention and control groups, and the SWC. For all calculations and procedures suggested in this review, we have provided instructions and resources in the Supplementary File to assist.

Finally, it is important to acknowledge the differences between combining procedures outlined to identify an intervention successful for an individual (e.g., true score change CI's and SWC, as demonstrated in SF-S9), and estimating the proportion of response in group-based interventions (SF-S11). With the former, there is no attempt to distinguish between intervention and non-intervention causes of change. In addition, the procedures outlined for the individual are heavily influenced by the relative magnitudes of measurement error and SWC. The approach described herein, requires that an individual's observed score change exceeds the SWC by, at least, the TE of the test. In scenarios where the TE is large, individuals will typically require true score changes substantially beyond SWC to identify an intervention as a success. Note, this conservative approach is required to routinely avoid individuals obtaining observed score changes greater than the SWC due to the randomness of measurement error alone. In contrast, the procedures described in section 3 to estimate proportion of response do distinguish between intervention and non-intervention causes of change. Estimating the proportion of response using this approach, is to some extent, less influenced by large measurement errors. This is due to the fact that the effects of measurement error are accounted for by variation observed in the control group and are thus removed from the final calculation. With greater participant numbers in the intervention and control group, estimates will become more precise and uncertainty reduced. As a result of these differences, it is possible that the proportion of individuals identified to experience a successful intervention (SF-S9), and the estimate of the proportion of response (SF-S11) will be different. Given the infrequent data collection points routinely used in practice (e.g., pre- and post-intervention), caution is required when interpreting at the level of individuals and it should be remembered that CI's are to be interpreted over the long-run. In scenarios where large measurement errors occur, practitioners/researchers can use knowledge of group-based estimates of response, to provide greater context when evaluating data observed from individuals.

## Conclusion

A personalized approach to sports nutrition is increasing in popularity due to recognition of the myriad of factors that influence individual response to nutrition and exercise related interventions. The presence of measurement error and biological variation renders identification of baseline values, change values and response status challenging, thus strategies to account for these issues have been proposed, enabling practitioners, and researchers to make informed decisions and judgements from the data they collect.

## Author contributions

ED and PS originally conceived the idea for this review. PS provided the statistical expertise and lead the writing of the review, along with the development of the Supplementary Files, with support from BH. Ongoing critical input was received from BS, BG, and ED. All authors read and approved the final manuscript.

### Conflict of interest statement

The authors declare that the research was conducted in the absence of any commercial or financial relationships that could be construed as a potential conflict of interest.

## Footnotes

^1^The calculation used to obtain a true score change is $\left(OS_{post}-OS_{pre}\right)\pm M\sqrt{2}$TE, where *M* is the multiple used to set the CI width. With (*OS*_*post*_ − *OS*_*pre*_)± TE we have set *M* to $\left(\frac{1}{\sqrt{2}}\right)=0.71$, and if we check the unadjusted multiples provided in Table 2, this approximately equates to a 50% CI.

## Supplementary material

A maintained version of this Supplementary File can be found at github.com/sportscientist.

## 4. Appendix I

For the calculation of CIs it is useful to introduce additional notation and concepts. The first is the notation: 100(1 − α)%, which describes the width of the CI. Here, α is a variable that we choose to set the interval and importantly link the width to the correct multiple of our TE estimate. For example, to set a 90% CI then α must be set to α = 0.1 to give 100(1 − 0.1)% = 90%. Given the consistent assumptions that observed scores are normally distributed we evoke the relevant properties of the distribution, such that a 100(1 − α)% CI for true score is obtained with *O*_*s*_ ± *TE* × *Z*_(1−α/2)_. The coefficient *Z*_(1−α/2)_ is referred to as the (1 − α/2)-th quantile of the standard normal distribution. In our example where we set α to 0.1 (i.e., for a 90% confidence interval), we require *Z*_(1−0.1/2)_, or the 0.95th quantile of the standard distribution. To obtain this value we can look up standard statistical tables or use software such as MS Excel. Using these methods, we find that *Z*_0.95_ is equal to 1.64 and so a 90% true sore CI for an individual would equal *O*_*s*_ ± *TE* × 1.64.

It is important to acknowledge that we can never definitively state the TE and studies only report imperfect estimates $\hat{\text{TE}}$, where accuracy will depend primarily on the number of individuals (or number of repeated trials) used in a test-retest. To account for this additional uncertainty, we use the (1 − α/2)-th quantile value from a t-distribution which is similar in shape to the normal distribution but has heavier tails (i.e., greater proportion of values away from the center). The specific t-distribution is based on numbers used in our TE estimate and we say that it has degrees of freedom equal to *n* − 1. In the data sets provided in this review, we include 20 participants (*n* = 20) to estimate TE from test-retests, and as such a 90% true score CI for each individual is equal to $O_{s}\pm \hat{\text{TE}}\times t_{19,\;0.95},\;$(i.e., the 0.95th quantile of the t-distribution with 19 degrees of freedom). Looking up statistical tables or use of software identifies that *t*_19, 0.95_ = 1.73 and so our 90% true score CI is calculated with $O_{s}\pm \hat{\text{TE}}\times 1.73.$ Alternatively, if we wanted to calculate a 50% true score CI with the t-distribution, we would set α = 0.5, *t*_19, 0.75_ = 0.69 to give $O_{s}\pm \hat{\text{TE}}\times 0.69$. What is important to note, is that as the number of individuals increases the t-distribution approaches the normal distribution such that the coefficients used to multiply the TE become similar.
